# Acceptance rates and reasons for social oocyte cryopreservation among women: systematic review and meta-analysis

**DOI:** 10.1007/s10815-025-03425-5

**Published:** 2025-03-01

**Authors:** Özden Tandoğan, Gözde Küğcümen, İlkay Güngör Satılmış

**Affiliations:** 1 Department of Nursing, İstanbul Arel University, Cevizlibağ Istanbul, Turkey 34020; 2 Department of Midwifery, School of Health Science, Istanbul Medipol University, Kavacık Istanbul, Turkey 34810; 3 Department of Women’s Health and Gynecologic Nursing, Istanbul University Cerrahpaşa Florence Nightingale Faculty of Nursing, Şişli Istanbul, Turkey

**Keywords:** Social oocyte cryopreservation, Social oocyte freezing, Acceptance, Systematic, Meta-analysis

## Abstract

The aim of this systematic review and meta-analysis was to determine the acceptance rates and reasons for social oocyte cryopreservation (SOC) in the general population (subgroup 1) and healthcare professionals/students (subgroup 2) according to the current literature. Relevant studies published between 2007 and Sept 2023 were identified from electronic databases, including PubMed, EBSCO MEDLINE Complete, Web of Science, Science Direct, Scopus, and CINAHL. Quantitative studies reporting women’s acceptance rates and reasons for social oocyte cryopreservation were eligible. A total of 20 quantitative studies were included in this process. Meta-analyses were conducted using random-effects models to evaluate study effect sizes. A total of 20 articles were analyzed. While the acceptance rate of SOC women in the general population was 56.5% (95% CI = 47.8–64.9%; *τ*^2^ = 0.98, df = 20; *p* < 0.001), this rate was between 42 and 66% in female healthcare professionals/students (*p* > 0.05; *I*^2^ = 98.46%; df = 20; *p* < 0.001). Among the acceptable reasons for SOC, cost-recovery (67.9%, 95% CI = 58–76%, *I*^2^ = 97.88%, *τ*^2^ = 0.95, df = 6; *p* < 0.001) and inability to find a suitable partner (45.7%, 95% CI = 32.6–59.5%; *I*^2^ = 97.96%, df = 10; *p* < 0.001) stand out. Acceptance rates did not differ significantly between the general population and healthcare professionals/students’ inability to find a suitable partner (*p* > 0.05). The meta-analysis shows that more than half of women accept SOC, with the acceptance rate increasing in the absence of a partner and if the cost is affordable.

**Trial registration** CRD42023455656

## Introduction

Oocyte cryopreservation, thanks to the techniques developed in the last three decades, has led to a significant increase in the number of cycles performed for various reasons, such as cancer patients [1]. This method is recommended and is used not only in cancer patients, but also to address age-related fertility loss, to prevent decline for medical reasons (e.g., endometriosis, preimplantation genetic screening), and in other clinical applications [2–4]. This study evaluates the acceptance rates of social oocyte freezing practices and the reasons why women prefer this method.

“As women age, their fertility capacity decreases” [4]. “This technique offers the possibility of freezing oocytes at a young age to increase the likelihood of becoming a mother in the future, making it a viable option for women with reproductive health issues and those who intend to delay childbearing rather than specifically seeking motherhood at an advanced age” [1]. The growing acceptance of social oocyte cryopreservation (SOC) among women presents challenges for health policy and traditional gender roles. Understanding the motivations behind this trend is crucial for advancing women’s health rights and gender equality [2].

Women’s motivations for choosing SOC include career planning, educational goals, health concerns, and partner status [5–7]. This highlights the importance of supporting women’s rights to expand their reproductive options in line with their lifestyles. While SOC is seen as a means for women to balance professional and maternal roles, it has also faced criticism. Some bioethicists argue that corporations may use SOC as a perk to encourage women to delay motherhood, enabling them to work longer hours for the benefit of the company, potentially undermining genuine gender equality [8, 9].

In recent years, there has been a significant increase in social oocyte cryopreservation (SOC) practices. For example, the acceptance rate in Sweden was 70% in 2016 [5] and 61.3% in Australia in 2023 [10.] This increase is attributed to women’s career and educational aspirations, while the low rate of 34% in Lebanon is explained by cultural and knowledge gaps [10]. This shows that social oocyte freezing practices are not only individual decisions but also a reflection of social changes. Increasing women’s awareness and reviewing health policies may contribute to the spread of these increases. Research indicates that women’s acceptance of SOC is influenced by personal, social, and economic factors [11–13]. Women with career aspirations are more likely to pursue this option to preserve their fertility potential. Evaluating these factors is essential to understanding and supporting women’s decision-making processes in this area [14].

Increasing acceptance rates of social oocyte freezing have important implications for health policies. Preserving women’s fertility potential may increase their likelihood of becoming a mother as they age, which may also have implications for population policies and birth rates [9, 14]. Health policies may therefore need to be reviewed with a view to increasing acceptance rates of social oocyte cryopreservation. A better understanding of the factors contributing to the increased acceptance of social oocyte cryopreservation may increase women’s awareness about preserving their fertility potential. Education and awareness-raising activities in this area may enable women to make more informed decisions. Existing research has significant gaps in terms of acceptance rates of social oocyte cryopreservation (SOC) practices and the motivations behind these practices. Studies on women’s attitudes towards SOC and the determinants of these attitudes are limited. In the literature, there is insufficient data on how acceptance rates of SOC vary among different demographic groups [2, 14–16]. In addition, the reasons why women prefer this method and the impact of factors such as economic factors, partner status, and social pressures have not been examined in sufficient depth. These gaps in knowledge pose significant barriers to the formulation of health policies and raising awareness of women about their fertility options. Therefore, this study was conducted to better understand the acceptance rates of social oocyte freezing practices and the motivations behind these practices. The acceptance and widespread adoption of social oocyte cryopreservation practices are directly related to women’s level of awareness and the reliability of information sources. Healthcare providers, media, and other information sources significantly shape women’s perceptions and awareness about SOC. [13, 14]. In this context, raising women’s awareness about SOC and ensuring their access to the right information sources can increase the acceptability of this method. Our research is critical to understanding women’s fertility preservation strategies and assessing their impact on society. It also aims to provide data for health professionals and decision-makers to better understand women’s attitudes and needs in this regard. This study will make an important contribution to the development of health policies and the defense of women’s reproductive rights.

*Study question:* The objective of this systematic review and meta-analysis is to determine the acceptance rates and underlying reasons for social oocyte cryopreservation (SOC) among women in the general population (subgroup 1) and female healthcare professionals/students (subgroup 2). The following research questions will be addressed:

1. What is the proportion of women who find social oocyte cryopreservation acceptable in women in the general population and female healthcare professionals/students in the world?
2. What are the acceptable reasons or situations for social oocyte cryopreservation in women in the general population and female healthcare professionals/students in the world?

## Method

A systematic review and meta-analysis were performed following the PRISMA guidelines. The study was registered to the PROSPERO International Prospective Register of Systematic Reviews (register number: CRD42023455656).

### Main outcome(s)

The objective of this systematic review is to determine the proportion of women who find oocyte freezing an acceptable option and to identify the reasons why they accept it.

### Participants/population

The selected studies included data from female participants in descriptive research where questions were asked about their agreement with and reasons for social oocyte cryopreservation. The participants consisted of women from the general population (subgroup 1) and female healthcare professionals or students (subgroup 2).

### Intervention(s), exposure(s)

Quantitative studies in which women were asked questions about their social oocyte freezing agreement and reasons were included.

### Comparator(s)/control

Comparison (C): Social oocyte freezing acceptors and non-acceptors were compared, and subgroup comparisons were made for the general female population and health professionals.

### Search strategy

To identify relevant literature for review, major medical electronic databases, including PubMed, Ebsco MEDLINE Complete, Web of Science, ScienceDirect, Scopus, and CINAHL, were searched between July and September 2023. The search terms were selected by combining different possible keywords, including “egg OR oocyte” and “cryopreservation OR freezing OR vitrification OR fertility AND preservation,” as well as “social OR elective” and “knowledge OR attitude OR intentions OR practice OR awareness OR acceptance.”

### Eligibility criteria

Studies reporting findings about our primary outcomes, including acceptance rates and reasons for social oocyte cryopreservation in women in the general population and female healthcare professionals/healthcare/medicine students, were eligible for inclusion without limitation on the publication year. All descriptive studies, including cohort studies, case–control studies, and cross-sectional studies, were eligible for inclusion. Qualitative studies were excluded from this review because they did not report acceptability rates of oocyte freezing among women, and those who had previously frozen oocytes were also excluded. The literature search was limited to papers published in English.

### Study selection

Following the removal of duplicate articles from disparate databases, two researchers (Ö. T., G. K.) conducted an initial review of the titles and abstracts to determine which studies satisfied the established inclusion and exclusion criteria. Those studies that met the inclusion criteria or could not be identified from the title or abstract were then reviewed in full. In cases where there was no consensus, the third researcher (İ. G. S.) was consulted. Rayyan software was employed to store, organize, and extract data. A total of 20 studies were deemed eligible for inclusion.

**Fig. 1:**
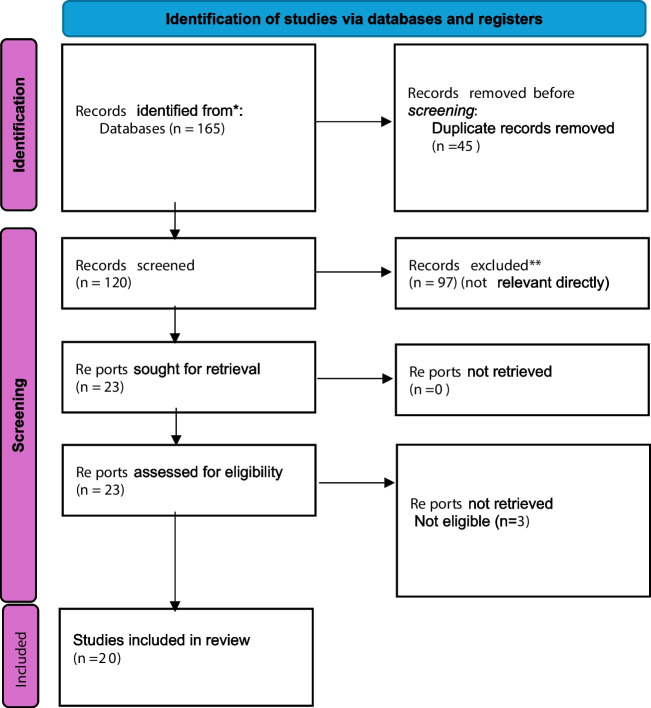
PRISMA 2020 flow diagram for new systematic reviews which included searches of databases and registers only. Asterisk (*) indicates to consider, if feasible, to do so, reporting the number of records identified from each database or register searched (rather than the total number across all databases/registers). Asterisks (**) indicate that if automation tools were used, indicate how many records were excluded by a human and how many were excluded by automation tools. *From:* Page MJ, McKenzie JE, Bossuyt PM, Boutron I, Hoffmann TC, Mulrow CD, et al. The PRISMA 2020 statement: an updated guideline for reporting systematic reviews. BMJ 2021;372:n71. https://doi.org/10.1136/bmj.n71

Figure 1 presents a PRISMA flowchart that provides a summary of the literature search and study selection process. A total of 165 articles were identified through the electronic database search and manual search. Following the removal of duplicate records (45), a total of 120 articles were deemed eligible for inclusion. The titles and abstracts were then screened to identify relevant articles, as well as review articles, protocols, duplications, or articles focusing on different populations. In total, 97 articles were excluded from the process because they did not meet the inclusion criteria. The remaining 20 full-text articles underwent a rigorous assessment for eligibility (see Fig. 1).

### Data extraction

A data extraction tool was developed by the research team to record the research data. Two researchers conducted independent data extraction using the provided template, which included the following variables: authors, country of study, study design, sample size, the proportion of women who had social egg freezing, and situations in which women find social oocyte freezing acceptable, stratified by the general community and healthcare professionals.

### Methodological quality

To avoid the potential for bias in the analysis of the studies’ statistics, all included studies underwent a comprehensive critical appraisal to ensure their validity. In order to assess the validity of the findings of the included studies, design-specific standardized quality assessment tools were employed for studies of differing designs. The Joanna Briggs Institute (JBI) checklists for cross-sectional studies, case studies, case series, and case–control studies were employed in the quality assessment of the articles (Table 1). The tool comprises nine items. Each item is evaluated according to the following criteria: Yes, No, Uncertain, and Inapplicable. The results of the assessment were presented in the form of a quality score, calculated on the basis of the total number of items considered (number of yes), in accordance with the methodology proposed by Aromataris and Munn [15].

**Table 1:** Characteristics of studies included in the systematic review and meta-analysis on women’s attitudes and intentions towards social ovarian freezing

	Full title of the study	Study/country/date	Study group, sample characteristics and number/data collection method/age	What is the percentage of women in the world who consider social oocyte freezing acceptable?	What are the situations in which women around the world consider social oocyte freezing acceptable?	Quality score
1	[17]	Stoop, Nekkebroeck, and Devroey/Belgium/2011	1024 women aged 21–40 years living in Belgium, electronic survey/potential oocyte freezers (*n* = 323) mean: 28.57 Doubtful group (*n* = 171) mean: 28.70 Non-freezers (*n* = 530) mean: 32.28	48.2%	If they have more confidence about the risks to their future fertility (75.2%) and the health safety of children derived from frozen oocytes (70.9%)	Yes: 9/9
2	[26]	Hee Hong et al./Korea/2019	71 women between the ages of 20 and 45 who want to freeze oocytes for social reasons/by filling out a form at a hospital in Seoul Mean: 37 20–24 (*n* 2) 25–29 (*n* 5) 30–34 (*n* 12) 35–39 (*n* 36) 40–45 (*n* 16)	71.8%	Wishing to preserve their future fertility (70.6%) If the cost of egg freezing is covered (61%)	Yes: 5/9
3	[5]	Wennberg et al./Sweden/2016	987 women between the ages of 30–39 living in Switzerland/by filling out the questionnaire sent by mail Total = 34.4 ± 2.8 National = 34.6 ± 2.8 Urban = 34.2 ± 2.8	70%	No	Yes: 8/9
4	[19]	Arendt and Oxlad/Australia/2023	514 women aged 18–44 years/by filling out the online survey form Mean: 27.32 ± 6.43 18–29 (*n* = 334) 30–34 (*n* = 98) 35–39 (*n* = 46) 40 + (*n* = 29) Age not supplied (*n* = 7)	61.3%	If financing is available 47%	Yes: 8/9
5	[18]	Petersen et al./Denmark/2015	340 women aged 35–43 years were examined in the clinic/by filling out a web-based questionnaire form Total = 37.4 ± 2.0 In a relationship = 37.2 ± 2.1 Single = 37.5 ± 2.0S	45%	No	Yes: 9/9
6	[23]	Anspach et al./USA/2017	53 medical students and house staff/by filling out a questionnaire form 20–25 = 17.0% 26–30 = 52.8% 31–35 = 26.4% 36–40 = 1.9% No response = 1.9%	Before the training, 54.3%, and after the training, 71.7%	No	Yes: 7/9
7	[22]	Nasab et al./USA/2019	350 medical doctors, students, and faculty members/by filling out the online survey form Female *n* 246 Male *n* 93 Total *n* 339 21–25 (*n* 61) 26–30 (*n* 106) 31–35 (*n* 72) 36–40 (*n* 42) 41–45 (*n* 17) 46–50 (*n* 13) 51–55 (*n* 6) > 55 (*n* 19) Total (*n* 336)	30%	Aging 32% Career plan 31% Absence of partner 20% Lack of a stable relationship 2%	Yes: 9/9
8	[8]	Daniluk and Koert/Canada/2016	500 women aged 18–38 years/by filling out the online survey form/28.5 ± 5.33	66%	Cost (85.6%) Own health risks (86.4%) or health risks to children (87.8%) Success rates (82%)	Yes: 9/9
9	[21]	Armstrong et al./USA/2023	122 medical students/by filling out the web-based survey form Mean: 25.20 ± 2.75	65%	Not having a suitable partner (51%) Probability of success (89%) Financial reimbursement (70%) Complexity of treatment (76%) Desire for children (87%) Child health (94%)	Yes: 9/9
10	[24]	Esfandiari et al./USA/2019	103 gynecology assistant/by filling out the online survey form 26–30 (*n* 68) 31–34 (*n* 29) ≥ 35 (*n* 6)	45.6%	If there is no partner (61.2%)	Yes: 8/9
11	[7]	Tozzo et al./Italy/2019	930 university students aged 18–35/by filling out the questionnaire form 18–22 (*n* 744) 23–26 (*n* 134) 27–31 (*n* 26) ≥ 31 (*n* 17)	19.5%	Enabling the woman to find the right partner (26.5%) Ensuring the woman feels ready for motherhood (26.5%) Women having economic stability (50.2%)	Yes: 9/9
12	[28]	Sayegh et al./UAE/2023	422 women aged 18–38/by filling out the online survey form 18–25 (*n* 195) 26–30 (*n* 64) 31–38 (*n* 163)	43.4%	If the baby born from the oocyte is confirmed to be healthy (37.6) If there are no risks to their health or future fertility (21.1%) If the success rate of achieving pregnancy is high (12.3%) If the cost of the procedure is covered (9.3%)	Yes: 9/9
13	[29]	Nunes et al./Portugal/2023	257 women aged 18–45 years/by redirecting to Google Forms through online advertisements Mean: 25.98 ± 6.06	14%	To prevent the effect of age on oocyte quantity and quality (56.3%) In the absence of a partner (26.3%)	Yes: 9/9
14	[33]	Woodtli et al./Switzerland/2018	248 women aged 15–35 years/by filling out the online survey form Mean: 25.1 ± 4.9 16–20 (*n* 37) 21–25 (*n*109) 26–30 (*n* 62) 31–35 (*n* 40)	19%	Not having a partner (30%) Career (16%)	Yes: 9/9
15	[20]	Johnston et al./Australia/2020	656 women aged 18–60 years/by filling out an anonymous online survey form with/a median of 28	65%	Not having a suitable partner (75%) Financial insecurity (72%) Career/educational advancement (65%)	Yes: 9/9
16	[30]	Santo et al./Brazil/2017	444 women of reproductive age/by filling out the online survey form via e-mail Mean: 33.2 ± 6.6	85.4%	No	Yes: 8/9
17	[25]	Ikhena-Abel et al./USA/2017	99 medical students/ by filling out the online survey form via e-mail Mean: 25.1 ± 2.7	71%	Not having a suitable partner (83%) Probability of success (95%) Child’s health (94%) Financial reimbursement (86%) Complexity of treatments (78%) Desire to have children (89%)	Yes: 7/9
18	[31]	O’Brien et al./ Ireland/2017	663 women aged 18–44 years/by filling out an online survey form via social media 18–24 (*n* 55) 25–29 (*n* 143) 30–34 (*n* 228) 35–39 (*n* 173) 40–44 (*n* 64)	72.2%	Postponed having a family for career purposes (18.5%) To preserve fertility (72.2%) In case of not having a suitable partner (40%)	Yes: 9/9
19	[27]	Lallemant et al./UK and Denmark/2016	973 women aged 18–68/by filling out the online survey form Median: 31 18–24 (*n* 207) 25–34 (*n* 412) 35–39 (*n* 136) ≥ 40 (*n* 186)	89%	Not having a suitable partner (32%) Postponing not to have a family for career (20%) Providing women who are not currently ready to have children with the opportunity to do so in the future (25%)	Yes: 9/9
20	[32]	Tan et al./ Singapore/2014	129 female medical students in Singapore/by filling out the electronic survey form Mean: 23.1 < 21 (*n* 2) 21–24 (*n* 91) 25–29 (*n* 35) > 30 (*n* 1)	48.9%	To postpone family planning for a career (45.7%) Not having a suitable partner (46.5%) In case of state support (71.3%)	Yes: 5/9

### Data synthesis and statistical analysis

A meta-analysis was conducted using Comprehensive Meta-Analysis software (Version 3.3.070) to determine the rates of social oocyte freezing. The rate of social oocyte freezing was calculated using a random-effects model. This method provides a more robust estimation of effect sizes. In employing the random effects model, each study is assigned a weight inversely proportional to its internal variance, thereby accounting for both within-study and between-studies variance. Accordingly, a random effects model is more appropriate for meta-analysis in the presence of heterogeneity. The degree of heterogeneity was quantified using Cochran’s *Q* and *I*^2^ statistics. The *Q* statistic is reported as a chi-squared value, and *p*-values are also provided. The *I*^2^ statistic is reported as a percentage. An elevated value of *I*^2^ indicates a greater degree of heterogeneity between statistics. For *I*^2^, 25%, 50%, and 75% correspond to low, medium, and high heterogeneity, respectively.

## Results

### Results of searches

The selected studies were quantitative research projects conducted in a variety of countries, including Belgium [17], Denmark [18], Sweden [5], Australia [19, 20], USA [21–25], Korea [26], Denmark/USA [27], Italy [7], United Arab Emirates [28], Portugal [29], Brazil [30], Canada[8], Ireland [31], Singapore [32], and Switzerland [33]. A total of 20 studies were included in this SRMA study. These studies were conducted between 2011 and 2023. The methodological quality of the studies was predominantly assessed as high-quality (5–9 points) in accordance with the JBI checklists.

The proportion of women who expressed acceptance of social oocyte cryopreservation ranged from 19 to 87% across all groups, from 17 to 89.7% in the general population, and from 17 to 87.7% among healthcare workers.

Reported reasons for increasing the acceptability of SOC were evaluated by grouping rates from studies with similar descriptions. The following categories were defined as follows: financial support for the procedure by insurance or an employer (covering the cost), not having a suitable partner for natural conception (inability to find a suitable partner), the desire to prevent age-related anomalies and have a healthy child when postponing pregnancy to an older age (the desire to have a healthy child), belief in the high success rate of the procedure (high success rate of the procedure), personal desire to become a mother and have children in her life (the desire to have children), and presence of career plans that hinder having a baby (career planning). As a result, the most prevalent acceptable reasons or situations for social oocyte cryopreservation were identified as covering the cost (58–76%), the inability to find a suitable partner (32.6–59.5%), the desire to have a healthy child (61–82%), the high success rate of the procedure (77%), the desire to have children (55–77%), and career planning (31%).

**Fig. 2:**
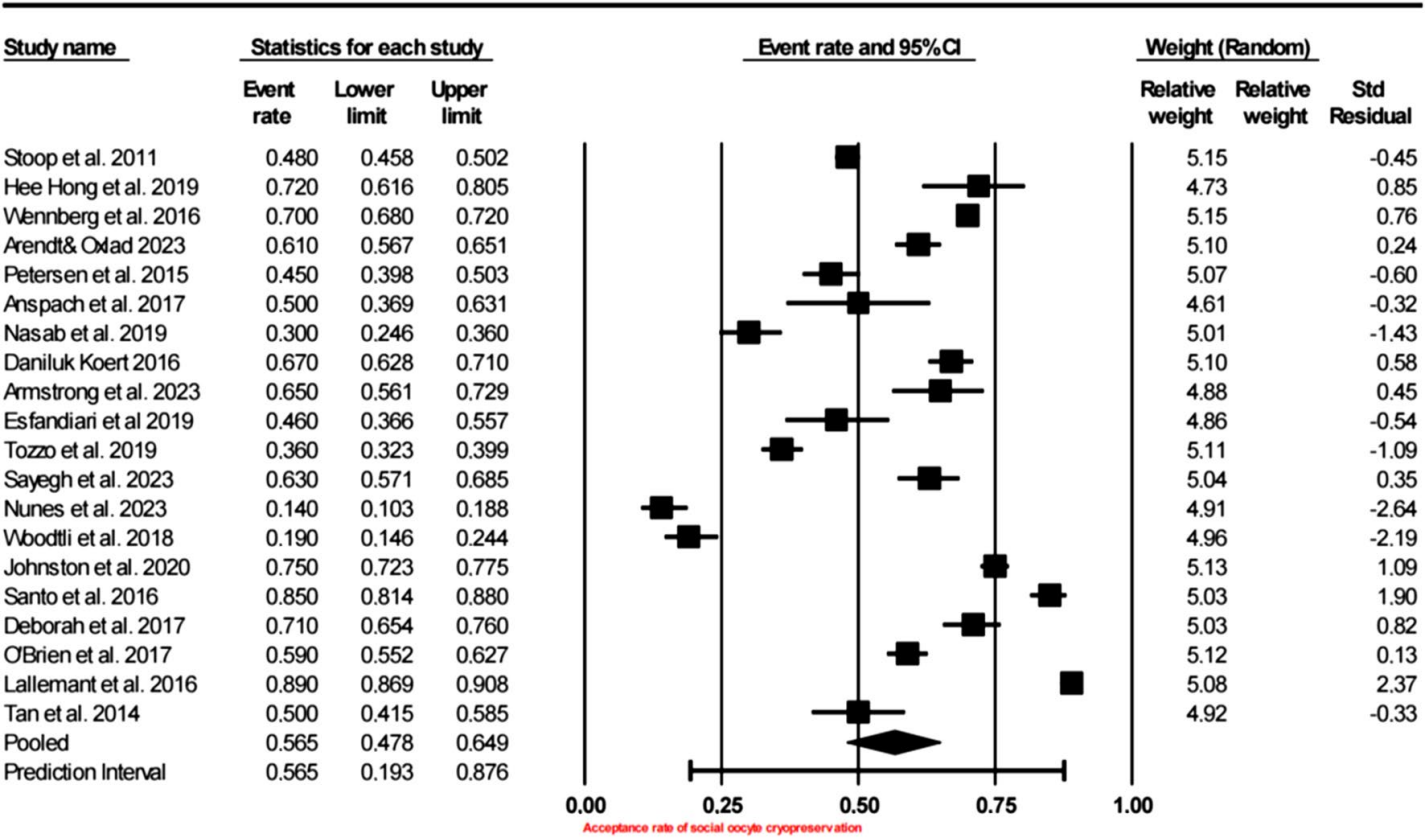
Acceptance rate of social oocyte cryopreservation

**Fig. 3:**
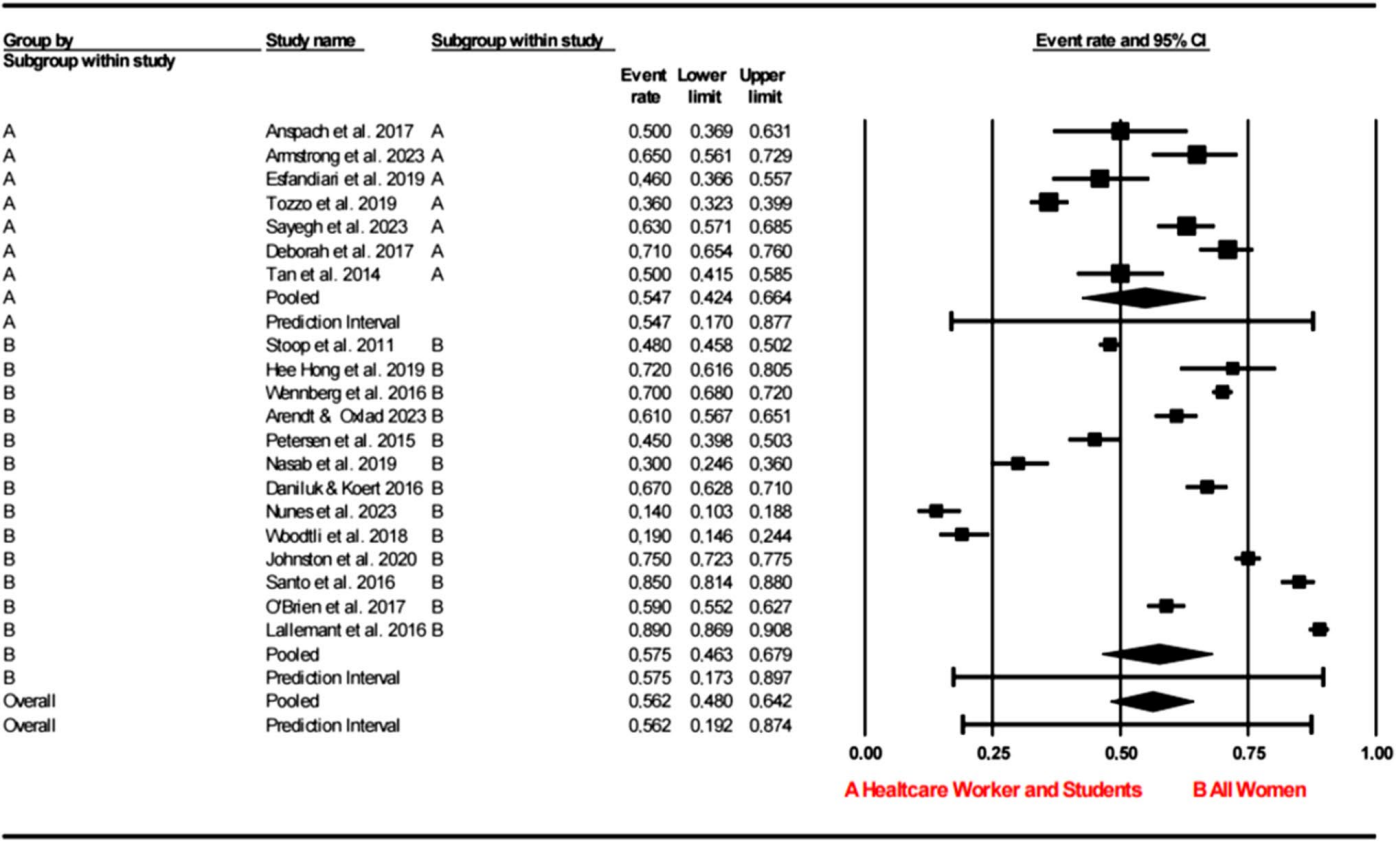
Health workers and students: acceptance rate of social oocyte freezing—subgroup analysis

The meta-analysis of the preceding research data revealed that the overall acceptance rate of social oocyte cryopreservation was 56.5% (95% CI = 47.8–64.9%) (see Fig. 2). A subgroup analysis of health workers/students and the general female population showed that acceptance rates were not significantly different (95% CI = A, health works/students 42.4–66.4 0.47%; B, all women 46.3–67.9%; *p* > 0.05). The heterogeneity was considerable with regard to the acceptance rates of social oocyte cryopreservation (*I*^2^ = 98.46%, df = 20; *p* < 0.001) (see Fig. 3). Effect size and 95% confidence interval, estimation interval, between-study difference statistics, and other heterogeneity statistics are also included in the table (see Fig. 6).

**Fig. 4:**
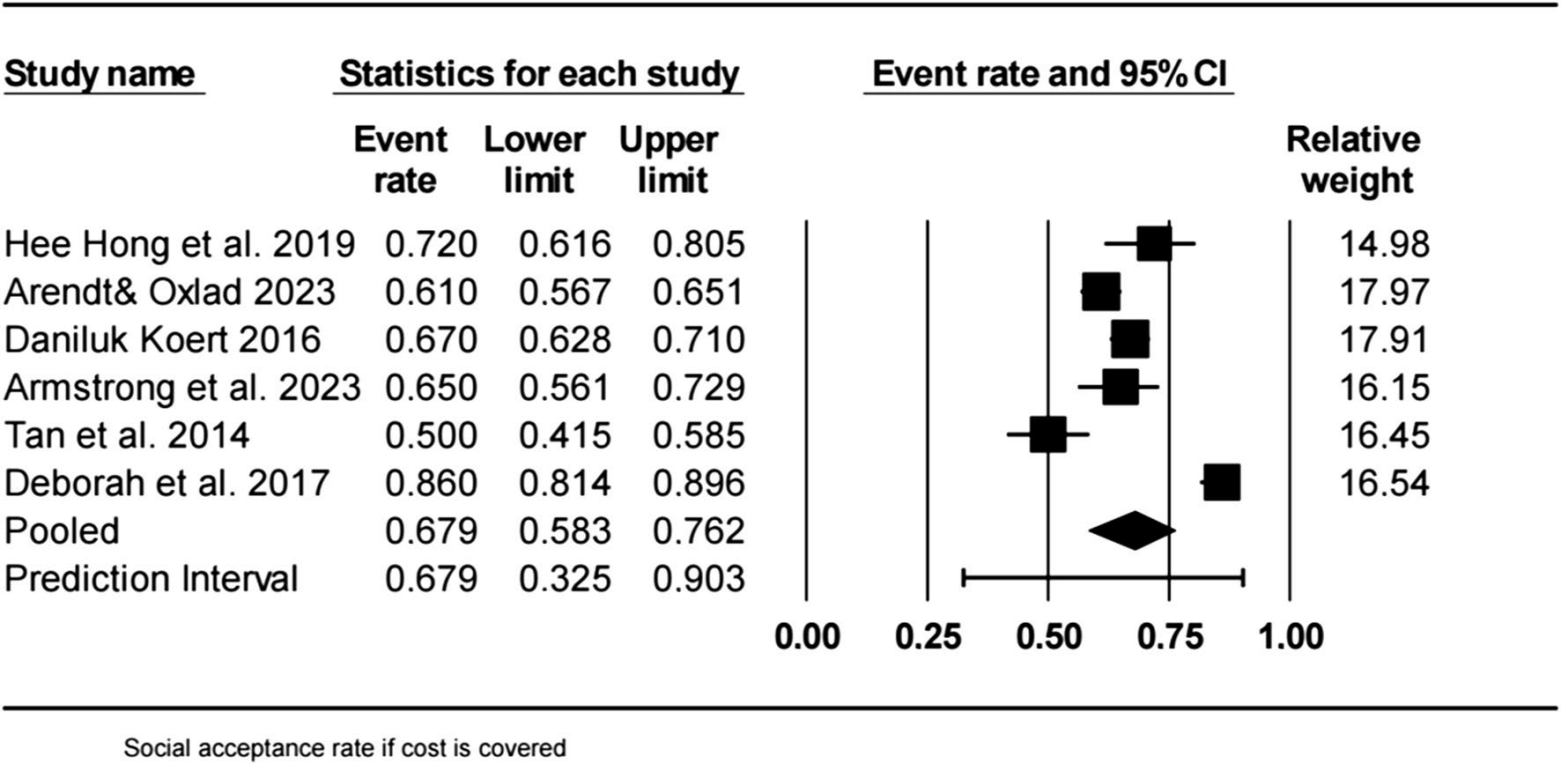
Social acceptance rate if cost is covered

The acceptance rates were evaluated in accordance with the most prevalent reasons for acceptance. The acceptance rate when the cost of social oocyte cryopreservation was covered was 67.9% (95% CI = 58.3–76.2%, *I*^2^ = 97.88%, df = 6; *p* < 0.001) (see Fig. 4).

**Fig. 5:**
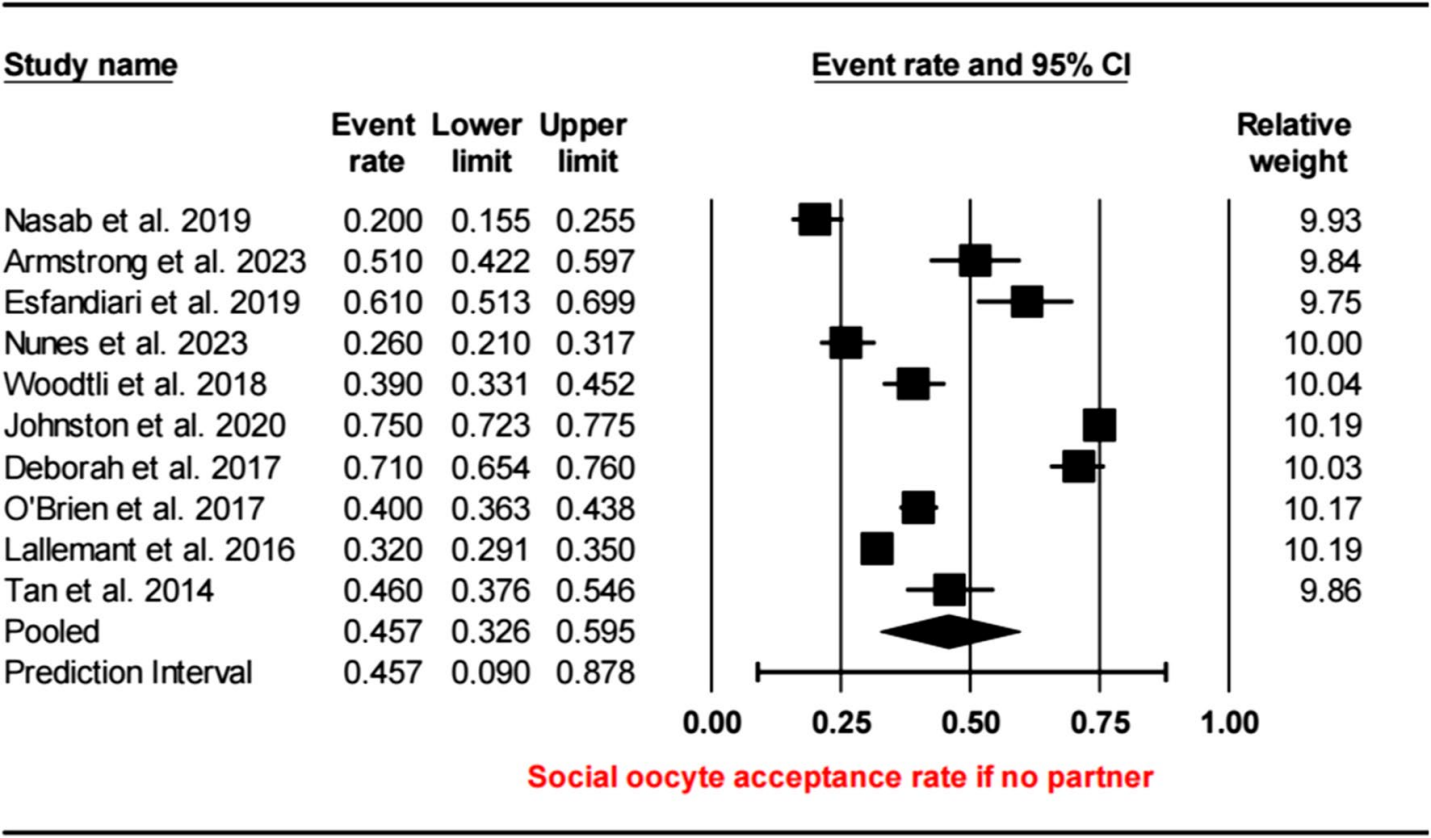
Social oocyte acceptance rate if no partner

**Fig. 6:**
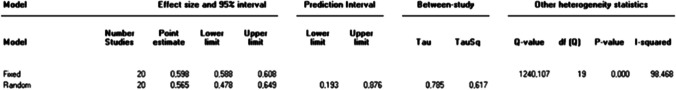
Heterogeneity of the acceptable rate of social oocyte freezing

Furthermore, in the absence of a partner, the rate was 45.7% (95% CI = 32.6–59.5%) (see Fig. 5). There was considerable heterogeneity in the data (*I*^2^ = 97.96, *τ*^2^ = 0.71, df = 10; *p* < 0.001). The acceptance rates, according to the reasons, did not differ significantly between the subgroups of the general population and healthcare professionals/students (coverage of cost, 95% CI = 0.59–0.45%; *p* > 0.05; absence of a partner, 95% CI = 0.51–0.55; *p* > 0.05) (Fig. 6).

## Discussion

This systematic review and meta-analysis comprehensively addressed acceptance rates and reasons for social oocyte freezing (SOF). The results demonstrate that SOF is widely accepted among women in the general population, female health professionals, and female students. The most significant finding of the study is that the overall acceptance rate of SOF is 56%. This indicates that SOF is becoming a more prevalent practice and that women view it in a favorable light. The most common acceptable reasons or situations for social oocyte cryopreservation were identified as affordability, inability to find a suitable partner, desire to have a healthy child, high success rate of the procedure, desire to have a child, and career planning. However, only two factors were meta-analyzed in this article. Considering that at least two articles are required for meta-analysis, it is clear that these factors should be addressed more comprehensively. Increasing the number of future studies and collecting more data is important for a more robust meta-analysis. In order to reduce the high heterogeneity (*I*^2^ = 98.67%), we performed subgroup analyses comparing acceptance rates between the general population and healthcare professionals/students. Subgroup analyses revealed no statistically significant difference in acceptance rates between the general population and health professionals/students. These findings suggest that SOC is accepted at a comparable level across diverse segments of society. However, the demographic diversity of the sample groups used in this study is limited. Our sample was restricted to certain age groups and occupational groups, so claiming that our results are valid for the whole population requires a cautious approach. The use of more diverse and representative sample groups in future research will allow a more comprehensive assessment of the acceptance rates of social oocyte freezing.

Research on social oocyte cryopreservation shows that this practice is accepted both in the general population and among health professionals [16, 21, 34]. In particular, the overall acceptability rate of 56% that you reported in your study provides interesting data when compared to other findings in the literature. Social oocyte freezing acceptance rates generally range between 60 and 80% [10]. The general acceptance rate of 56% obtained in our study is important in terms of understanding how much social oocyte cryopreservation is accepted in society and its possible effects on future practices. This rate indicates that individuals do not have sufficient knowledge about social oocyte cryopreservation and that more education and awareness raising are needed. In a study conducted in 2023, the acceptance rate of social oocyte cryopreservation among Lebanese women was 34% [10]. This low rate may be due to cultural and social factors as well as women’s lack of sufficient knowledge on this subject. The traditional family structure and social norms in Lebanon may especially create resistance against such practices. In a study conducted in Sweden, the acceptance rate of social oocyte freezing was found to be 70% [12]. This increase can be explained by the effect of increased awareness and education levels in the society. Social support systems and health policies in Sweden facilitate women’s access to these services. In another study conducted in Denmark and the UK, this rate was around 89%. This high acceptance rate reflects the strong health policies and social support systems in these countries, as well as women’s search for flexibility in career planning and family formation. In terms of their place in the literature, these studies expand the existing knowledge on social oocyte cryopreservation and provide an important resource by comparing acceptance rates in different countries. Furthermore, their relationship with previous research provides a critical basis for understanding trends and changes in this area. This suggests that social oocyte cryopreservation is accepted among women, but cultural and socio-economic factors also influence this acceptance rate. It also highlights the importance of awareness and education efforts on social oocyte cryopreservation.

Social oocyte cryopreservation is an increasingly preferred method for women to preserve their fertility; however, the process may not be accessible to many women due to its high costs. A 2023 study indicated that the utilization rate of social oocyte cryopreservation is low, which has an adverse impact on its cost-effectiveness [19]. The study revealed that the utilization rate of frozen oocytes was approximately 16%, indicating that the financial burden of the high costs associated with this option makes it unappealing to many women [21]. Moreover, recent data indicates that fertility clinics have increased their storage fees, thereby further increasing the overall cost of social oocyte freezing [35, 36]. This restricts women’s access to this service, demonstrating that despite increasing awareness and demand for social oocyte cryopreservation, high costs constitute a significant barrier [9]. Consequently, social oocyte cryopreservation should be regarded not only as a medical solution but also as a crucial economic decision, given the significant financial implications involved, which often make it challenging for women to contemplate their future fertility options. The results of this meta-analysis indicate that 68% of women would respond favorably to oocyte cryopreservation if the associated costs were covered. The removal of economic barriers may facilitate greater consideration of this option by a larger number of women.

The medical acceptance of oocyte cryopreservation is more prevalent than its social acceptance [2]. However, the lack of a suitable partner has also become increasingly prevalent, representing a significant rationale for women to pursue social oocyte cryopreservation [37]. This topic has been extensively discussed in the literature and is identified as a significant social obstacle that women encounter in their pursuit of expanded reproductive options. In various studies, women have indicated that their consideration of this option is motivated by a desire for a stable relationship and the desire to have children in the future. For example, one study reported that the inability to find a suitable partner had a direct influence on women’s decisions regarding oocyte freezing [37]. This method of fertility preservation is preferred by women in the event of uncertainty in their current relationship or inability to find a suitable partner [38]. Furthermore, the desire to maintain one’s social position is also a factor influencing women’s decisions regarding oocyte freezing.

Social oocyte cryopreservation has emerged as a significant strategy for women seeking to preserve their fertility. In this context, the most frequently cited reasons were cost and the inability to find a suitable partner (32–59%). A review of the current literature on this issue reveals that women’s decision-making is influenced by a multitude of social and psychological factors, as well as uncertainties inherent to their relationships [21, 23, 39]. For example, one study describes how women shape their oocyte-freezing decisions due to their inability to find a suitable partner and that many women are unable to find an appropriate partner with whom to have a child [8]. Another study examines the motivations behind women’s decisions to freeze oocytes for social reasons and discusses the role of the search for a suitable partner in this process [23, 39].

The lack of a suitable partner is an important factor shaping women’s attitudes towards social oocyte cryopreservation, as well as the lack of adequate information about it [9]. In this study, more information about social oocyte cryopreservation may enable women to make informed decisions in this process. “Lack of a suitable partner” is an important social factor affecting women’s decisions about social oocyte cryopreservation, and this plays a critical role in women’s assessment of their future fertility options.

The present study revealed no statistically significant differences in acceptance rates between the general population, health professionals, and students. These findings indicate that SOC is accepted at a comparable level across different segments of society. This suggests that health professionals and students may not possess a distinct perspective compared to other societal segments and that their needs and expectations may be analogous. Those engaged in health-related professions and pursuing academic studies in the field of health are typically more informed and educated about the range of health services and social assistance available to them. Nevertheless, the comparable acceptance rates indicate that, despite this awareness, social issues and requirements are addressed with a shared understanding within the community [21].

Health care providers, media, and other information sources significantly shape women’s awareness and perceptions about social oocyte cryopreservation. Women’s level of knowledge and the reliability of information sources are important factors influencing their decision to undergo social oocyte cryopreservation. In Esfandiari’s study, although reproductive endocrinology and infertility courses included almost all specialists trained in egg freezing, only half of the female specialists felt comfortable consulting with their patients on this topic [24]. In the future, it is critical that all doctors, not just infertility specialists, have a more comprehensive knowledge of family planning so that people feel more confident in counseling on this topic [40]. In Akdondi’s study, postgraduate students also expressed similar views about SOC. It is extremely important that both future doctors and women receive regular and effective education on reproduction, family planning, and egg freezing [41]. The extent and accuracy of the information provided by healthcare professionals about social oocyte cryopreservation may determine women’s attitudes towards this method. Similarly, the quality of information in the media and other popular sources may also shape women’s perceptions. Therefore, a detailed examination of women’s information sources and information quality about social oocyte cryopreservation will contribute to a more comprehensive understanding of the subject. Thus, it will be possible to develop educational and awareness-raising activities that will enable women to make more informed decisions.

## Conclusions

Consequently, the widespread acceptance of SOC practices in society will facilitate access to this method, thereby increasing women’s awareness of the importance of protecting their fertility potential and contributing to significant strides towards gender equality. Therefore, it is imperative that health policies are reviewed and that social oocyte freezing practices are supported. Partial or full coverage of the costs of social oocyte freezing by health insurance will increase women’s access to this method and support equal opportunities. In addition, organizing training and information campaigns to increase the awareness of health professionals and the public on this issue would also be an important step.

### Limitations, reasons for caution

The sample sizes of some included studies may not be large enough to ensure the reliability of the results. Additionally, the demographic diversity of the sample groups in this study is limited, as they primarily consist of specific age and occupational categories. This limitation may affect the generalizability of the findings across different segments of society. Furthermore, the inclusion of various study designs (e.g., cross-sectional, cohort) may complicate the comparability of results.

Qualitative research allows us to gain a deeper understanding of individuals’ decision-making processes, their motivations, and the social, cultural, and economic factors that are influential in this process. Therefore, the exclusion of qualitative data narrows the scope of the findings of our study and ignores a broader perspective on this issue. The inclusion of qualitative data in future research will enrich our understanding of this issue and allow us to better understand women’s decision-making processes.

## Data Availability

Data from this study will not be shared so that other researchers can conduct further analysis. There are some restrictions on sharing data; therefore, specific data will only be provided upon request. The data underlying this article will be shared on reasonable request to the corresponding author.
